# There’s more to food store choice than proximity: a questionnaire development study

**DOI:** 10.1186/1471-2458-13-586

**Published:** 2013-06-17

**Authors:** Rebecca A Krukowski, Carla Sparks, Marisha DiCarlo, Jean McSweeney, Delia Smith West

**Affiliations:** 1 Department of Preventive Medicine, University of Tennessee Health Science Center, 66 N. Pauline St, Memphis, TN, USA; 2 Office of Community Based Public Health, Fay W. Boozman College of Public Health, University of Arkansas for Medical Sciences, 4301 W. Markham St. #820, Little Rock, AR, USA; 3 Fay W. Boozman College of Public Health, University of Arkansas for Medical Sciences, 4301 W. Markham St. #820, Little Rock, AR, USA; 4 College of Nursing, University of Arkansas for Medical Sciences, 4301 W. Markham St. #529, Little Rock, AR, USA

**Keywords:** Food store, Health promotion, Obesity, Diet, Questionnaire

## Abstract

**Background:**

Proximity of food stores is associated with dietary intake and obesity; however, individuals frequently shop at stores that are not the most proximal. Little is known about other factors that influence food store choice. The current research describes the development of the Food Store Selection Questionnaire (FSSQ) and describes preliminary results of field testing the questionnaire.

**Methods:**

Development of the FSSQ involved a multidisciplinary literature review, qualitative analysis of focus group transcripts, and expert and community reviews. Field testing consisted of 100 primary household food shoppers (93% female, 64% African American), in rural and urban Arkansas communities, rating FSSQ items as to their importance in store choice and indicating their top two reasons. After eliminating 14 items due to low mean importance scores and high correlations with other items, the final FSSQ questionnaire consists of 49 items.

**Results:**

Items rated highest in importance were: meat freshness; store maintenance; store cleanliness; meat varieties; and store safety. Items most commonly rated as top reasons were: low prices; proximity to home; fruit/vegetable freshness; fruit/vegetable variety; and store cleanliness.

**Conclusions:**

The FSSQ is a comprehensive questionnaire for detailing key reasons in food store choice. Although proximity to home was a consideration for participants, there were clearly other key factors in their choice of a food store. Understanding the relative importance of these different dimensions driving food store choice in specific communities may be beneficial in informing policies and programs designed to support healthy dietary intake and obesity prevention.

## Background

Obesity is one of the most pressing public health problems, largely because it is a risk factor for a wide range of chronic diseases [1]. In recent years, efforts to understand the obesity epidemic have focused attention on understanding the role of the “built environment” (i.e., the physical surroundings that can impact dietary intake or energy expenditure, including food stores) [2]. Many experts now agree that the built environment must be considered in efforts to address obesity [3].

In some research, proximity of food stores, specifically supermarkets, has been positively linked with healthful dietary intake [4,5], and obesity [6-8], yet in other research, no association or negative associations have been found between the proximity of food stores and dietary intake [9-12] or obesity [11,13-15]. Perhaps helping to explain these inconsistent findings related to food store proximity and diet/ obesity, recent research has found that individuals frequently shop at stores that are not the most proximal [15-17] and little is known about the reasons which do guide selection of a food store. With the underlying assumptions that food store preferences influence food store choice, and that food store choice then proximally impacts dietary intake and more distally impacts weight status, it is important to understand how individuals select the stores at which they shop in order to design food store interventions that could alter dietary intake and weight status. There have been a few studies in the United States that have utilized short (i.e., five to seven item) measures to understand reasons for food store choice among low-income residents [18,19] or among Latinas [20]; however, a more comprehensive description of the factors influencing food store choice in a broader sample of individuals has not yet been conducted. In addition, the degree to which the questionnaires used in these studies were developed based on community input is uncertain, so it is unclear as to whether these questionnaires accurately represent the diversity of community perceptions and capture the full range of factors relevant to food store selection.

Obtaining a clear picture of the reasons associated with food store choice has been difficult up to this point because of the lack of a comprehensive measure which gathers information on all of the potentially relevant factors. Thus, the first phase of this study was to develop a descriptive questionnaire regarding the importance of key factors on food store selection which would be appropriate for use in a range of communities. A second phase was to field-test the questionnaire to gather initial information about the relative importance of various factors in food store choice in a diverse group with significant representation of populations at high risk for obesity.

## Methods

### Development of the food store selection questionnaire

Development of the Food Store Selection Questionnaire (FSSQ) involved a multi-step process including: multidisciplinary literature review of previous research examining food store choice, qualitative analysis of key themes emerging in community-engaged focus groups [21], review by a panel of experts, and review by community members. The questionnaire was developed with the goal that it could be used in a broad range of communities (e.g., urban/rural) to identify and compare the key factors in community members’ food store choice.

First, in March-May 2010, we reviewed current multidisciplinary literature using the PubMed database with the search terms of “food store,” “grocery store,” and “supermarket,” each combined with “choice,” “selection,” and “reason” to identify potential factors associated with food store choice in previous research. The references were then examined in the relevant articles, in order discover other potentially relevant articles. Based on the literature review, a list was created of factors that were previously cited as important in food store choice or were hypothesized to affect food store choice. This list was supplemented by items generated from key themes that emerged in 5 focus groups (in four communities) conducted in June-November 2010, in both rural and more urban settings with Caucasian and African American shoppers (n = 48), as described in detail elsewhere [21]. Participants were recruited through established networks of community organizations, assisted by a community liaison. The investigators employed the qualitative techniques of content analysis and constant comparison in interpreting the data [22,23]. Code words were assigned to relevant sections of data; related coded segments were combined into larger blocks of data and then into themes. These four main themes included proximity, financial considerations, food availability/quality, and store characteristics and had some, but not total, overlap with items reported in the literature. From this list of possible reasons for choosing a food store, a draft questionnaire was created.

An expert panel of individuals (n = 8) who work in the academic setting in the area of community-based research, in the fields of food environment assessment, survey methods, and health disparity research, reviewed the draft questionnaire. Three of the experts work in major metropolitan areas, and five conduct research in more rural areas. They reviewed the questionnaire to identify any missing key factors that may impact store choice, discern whether any items were redundant, and to nominate items for deletion. They were asked whether the questionnaire could be improved to enhance clarity of the items, instruction, or response scale. Experts’ feedback mostly related to addition of specific items and clarification of questionnaire instructions and items. The draft questionnaire was revised based on this feedback.

Next, the research team piloted the questionnaire with community members from across Arkansas using cognitive interviewing methods. Individuals (n = 12) who indicated that they were 18 years of age or older, the primary food shopper for a household of at least two individuals and not following a particular diet that requires shopping at only one store were asked to complete the questionnaire and then comment on the content and structure of the questionnaire instructions, items, and the response scale. They were asked to indicate whether the questionnaire was missing any key aspects of food store choice or whether items should be excluded. Instructions, items, and response scales that were identified as problematic or not well understood were reworded to improve comprehension and items were added and deleted. Saturation was reached (i.e., no new information was emerging) with twelve participants and interviews were discontinued. These community members were given a $20 gift card to offset the time and expense associated with participating in the cognitive interviewing. After verifying that the reading level of the revised questionnaire was below the eighth grade level, a final version of the questionnaire was created.

### Questionnaire administration sample

Participants for the questionnaire administration were recruited from seven communities (range: 10–19 participants from each community) across Arkansas through established networks of community organizations and agencies, facilitated by the participation of a community liaison from the University of Arkansas for Medical Sciences, College of Public Health’s Office of Community-Based Public Health in every stage of this research. We employed a multi-component recruitment approach that incorporated several methods, including: 1) direct, community-based efforts using small media (e.g., posters in local businesses, talks to local community groups, notices in churches, local newsletters); and 2) targeted invitations to known community gatekeepers likely to have access for dissemination to potentially eligible participants. To participate, an individual had to be: a) at least 18 years old, b) the primary food shopper for a household with a minimum of two individuals, and c) not following a particular diet that requires shopping at only one store due to specific availability of food items (e.g., gluten intolerance). Only one member of a household could participate. Communities were selected to reflect a range of constituencies, including urban and rural communities and racial diversity.

### Data collection

Questionnaire field testing occurred in groups at times identified as convenient for participants (i.e., evenings and weekends) in community locations (e.g., community centers, libraries, private rooms in restaurants, churches). After written informed consent was obtained, participants completed the FSSQ and questionnaires regarding sociodemographic data (i.e., age, gender, education level, employment status, racial and ethnic identification, marital status, receipt of Supplemental Nutrition Assistance Program or Women, Infant, and Child benefits), and food shopping behavior (e.g., typical number of stores visited each food shopping trip; form of transportation to the store). The FSSQ consisted of 63 items. Participants provided their perspectives on the importance of these items using two methods: first, they rated each item as to its importance in their choice of a food store on a five-point scale from 1 (not at all important) to 5 (very important); and then, on the same questionnaire, they were asked “Which of the previous reasons are the two most important reasons for you in choosing in a food store (briefly indicate item and item number)?” Measured body weight and self-reported height were then collected for all participants, and body mass index (BMI; weight [kg] / height [m]^2^) was calculated. Home ZIP codes were used to classify participants as living in a metropolitan area core (> 50,000 population) or a non-metropolitan area core (< 49,999 population) using the Rural Health Research Center’s ZIP code-level rural–urban commuting area codes 2.0 [24]. All participants received a $20 gift card for completion of the questionnaire.

The study was approved by the institutional review board at the University of Arkansas for Medical Sciences. Written informed consent was obtained from all participants.

### Data analyses

Descriptive statistics (frequencies, means and standard deviations) were calculated for participant characteristics and for each questionnaire item. Frequencies were utilized to characterize the number of times each item was chosen as one of the top two reasons for choosing a food store. Inter-item correlations were computed to identify items that may have high intercorrelations and were reviewed for redundancy. When the intercorrelations were suggestive of redundancies, we examined the items to determine which items, if any, were the best candidates for removal. Data were analyzed using SPSS 17.0 (2008, SPSS Inc., Chicago, IL).

## Results

### Questionnaire field test sample

Of the 139 individuals screened, 100 participated in the questionnaire field test. Reasons for non-participation included ineligibility (n = 6), a scheduling conflict (n = 8), no longer interested (n = 2) and scheduled but failed to attend (n = 23).

The sample (n = 100) was predominately female (Table 1). Eleven percent of the participants were normal weight, 28% were overweight, and 61% were obese. The sample was diverse with respect to age (range: 25–77 years), race, education level, marital status, and employment, and rurality.

**Table 1:** Participants’ (n = 100) sociodemographic characteristics and food shopping behaviors

***Characteristic***		***Mean (SD)***	***%***
Gender (% female)			93
Age (years)		50.6 (12.9)	
Race			
	African American		64
	Caucasian		35
	Asian		1
Ethnicity (% Hispanic)			0
Education (% completed)			
	Less than high school		4
	High school/GED		14
	Some college		36
	College		23
	Graduate degree		23
Employment			
	Full-time		58
	Part-time		10
	Retired		15
	Homemaker		6
	Unemployed		5
	Student		3
Marital status			
	Married		48
	Divorced/separated		29
	Widowed		6
	Never married		17
Rurality of residence (% non-metropolitan ZIP codes)			35
BMI		33.4 (8.2)	
Number of stores visited on a typical shopping trip			
	One		33
	Two		44
	Three		16
	Four or more		7
Location from which one departs for the food store			
	Home		92
	Work		8
Transportation to store			
	Public transportation		1
	Drive self		92
	Ride with other		5
	Walk		2
Participate in Supplemental Nutrition Assistance Program			19
Participate in Women, Infants, and Children program			4
Decision maker for food store choice			
	Self		93
	Family member		7
Satisfaction with food store			
	Very dissatisfied		2
	Somewhat dissatisfied		4
	Somewhat satisfied		31
	Mostly satisfied		36
	Very satisfied		27

### Item reduction

In considering items for the final version of the questionnaire, individual items were examined for their mean score and correlation with other items. As detailed in the List of Omitted Items, 13 items were omitted with a mean score less than 3 (i.e., “somewhat important” on the rating scale) and which were not identified as important by the literature review, focus group participants, or expert reviewers. Three items (i.e., proximity to public transportation, internet ordering, home delivery) with low mean scores were retained because investigators were concerned that the insufficient availability of these options in the relatively rural region in which the questionnaire was administered precluded determination that these factors were unrelated to selection of food store for other locations in which these options are available. One other item (i.e., food for religious reasons) was retained because of the concern that the largely Christian communities in which the questionnaire was administered in this study may not have had the religion-based dietary considerations (e.g., kosher, halal) that other communities may have. Finally, in examining the correlations between items, one item was omitted because of a high correlation with all of the other items related to location/proximity. All of the items included in the 49-item final version are listed in Table 2, and the final questionnaire is available in Additional file 1. 

### **List of omitted items**

Omitted for Low Mean Score

I like the music at the store.

It’s close to a school that family members attend.

I like that the store contains an ATM (that is, a cash machine) or bank.

The store has free samples of food available when I shop.

I see people I know when I go shopping there.

Other people who are like me shop there.

I like the architecture (that is, the building design) of the store.

The store has precooked or “ready to eat” foods that I like.

I like that the store is locally owned.

The employees at the store know me.

I like that the store is a national chain supermarket/grocery store.

My family prefers the store to others.

The store accepts WIC or food stamps as payment.

Omitted Based on High Correlation with Other Items

It’s on my way to another place that I regularly go.

**Table 2:** Ratings of importance for items food store selection questionnaire: final version

**Item number (final version)**		**Mean**	**SD**	**Frequency as top two factors**
9	I think the meat is fresh.	4.74	0.69	13
33	The store is well-maintained.	4.68	0.68	3
10	I can buy the kinds of meat that I want.	4.67	0.79	0
29	I think the store is clean.	4.67	0.65	13
30	I feel safe when I go there.	4.66	0.70	3
32	I am able to easily find the items on my shopping list.	4.63	0.71	2
7	I think the fruits and vegetables are fresh and not bruised.	4.59	0.81	16
8	I can choose from a wide variety of fruits and vegetables.	4.56	0.74	13
31	The store is open when I like to go shopping.	4.56	0.86	7
49	The store has a payment option that works for me (that is, personal check, type of debit/credit card, purchase account).	4.49	1.16	6
46	I think the store has good sales.	4.48	0.99	6
44	I think the store has low prices.	4.45	0.85	27
42	The store generally has enough cashiers open when I am shopping.	4.44	0.79	1
12	I can find the brands that I like.	4.38	0.83	3
27	I am familiar with the store and its layout.	4.37	0.90	2
18	The store has foods that are not going to quickly expire.	4.3	1.10	5
35	The employees at the store quickly respond to my needs.	4.26	1.00	3
14	The store has a good variety of healthy foods.	4.21	1.04	7
34	The store is kept at a good temperature.	4.19	1.00	0
13	I can buy lower cost items like store brand items.	4.17	1.12	3
45	The store accepts coupons.	4.16	1.20	2
26	I can always get a shopping cart/basket.	4.15	1.22	0
24	I can complete my shopping quickly.	4.07	1.07	5
25	I can easily find parking.	4.04	1.15	0
2	It’s close to my home.	3.92	1.12	22
41	The store has disinfecting wipes for the grocery cart available.	3.89	1.28	1
28	The aisles of the store are wide enough.	3.88	1.21	0
48	The store sends out a sale paper or advertisement that helps me plan what I will buy.	3.81	1.46	1
47	I can get a discount by using the store’s shopper’s card.	3.73	1.51	4
21	I like the size of the store.	3.69	1.29	2
43	The store generally has someone available to bag my groceries.	3.67	1.38	
40	The store treats their employees well (for example, pay and benefits).	3.65	1.39	0
19	The store has foods that I know how to prepare.	3.61	1.29	0
39	The store is environmentally-conscious.	3.59	1.36	0
15	I can buy the foods that my family needs for medical reasons (for example, low-salt or gluten-free foods).	3.58	1.49	0
6	I can buy locally grown/raised foods.	3.49	1.47	4
38	I like that the store has a bakery.	3.39	1.42	0
20	The store has foods that I grew up eating.	3.37	1.32	0
22	I like that the store has a butcher.	3.36	1.49	0
17	I can buy other non-food items I need (for example, clothing).	3.13	1.43	0
11	I can buy foods in bulk or large volumes.	3.06	1.36	0
23	I like that the store contains a pharmacy.	3.03	1.54	1
1	It’s close to my work.	2.99	1.35	2
3	It’s close to other stores where I shop.	2.97	1.26	0
5	I can buy organic/chemical-free foods.	2.91	1.55	3
16	I can buy the foods that I eat for religious reasons (for example, kosher).	1.75	1.34	0
4	It’s close to public transportation such as a bus, train, or subway.	1.6	1.16	0
36	I am able to order my groceries from this store on the Internet.	1.4	0.94	1
37	The store delivers to my home.	1.34	0.88	0

### Results from the field-testing of the FSSQ

Data from the finalized version of the FSSQ were examined. The importance of each item in driving food store choice for the individuals in the sample as a whole was examined by item mean and standard deviation as well as the frequency that the item was cited as one of the two top factors (Table 2). The items with the highest mean scores were also often frequently cited as a top factor, indicating consistency between these two approaches to characterize the salient factors driving food store selection.

## Discussion

Although there are many measures of the store food environment, one thing that has been missing from the food environment literature has been a comprehensive questionnaire that details the reasons for choosing one’s primary food store. The initial findings from the newly developed and field-tested FSSQ clearly indicate that there are several key factors related to food store choice and the data shed light on the relative importance of various reasons for choosing a food store among participants from diverse backgrounds.

These findings demonstrate important similarities and differences with previous research as to the key factors in food store choice. Several previous studies have demonstrated that isolated factors were relevant to store selection (i.e., safety, food quality, variety of foods, ability to complete shopping quickly, prices, proximity to home/work, store cleanliness, customer service, and store hours) [18-20]; however, none of the previous studies included all of these key items simultaneously and often distinct items were grouped together in a single question (e.g., “cleanliness and good service” or “it is cheap, has bulk items, and double coupons”). The emergence of some of these factors as among the most important ones identified as driving store choice, on a measure which included all these factors, is one of the main contributions of this research to the field because it demonstrates relative prioritization of the factors. In addition, while there was not an item in the current study specifically focused on cultural or ethnic foods that is directly comparable to the items used by Wang [19] and Ayala [20], the FSSQ has several items including “the store has foods that I grew up eating,” “the store has foods that I know how to prepare,” and “I can buy foods that I eat for religious reasons” that likely tap into a similar construct. In contrast to Wang and Ayala‘s research, none of these items were among the items indicated as most important in the current sample when provided with an extended listing of reasons for selecting a food store.

Because this questionnaire was developed through a multi-stage process that included considerable community involvement, the FSSQ is a more comprehensive measure than previously available. Specifically, several reasons that were cited as among the most important reasons for food store choice (e.g., brands, payment options) have not previously been included in research in this area [18-20]. In addition, it is a strength of this questionnaire that two different methods (i.e., 1–5 rating of importance, rating of top two reasons) for evaluating factors most important in food store choice are possible because one method may be more appropriate for a particular study than the other method. For example, for a community level-intervention, it may be considered to be more helpful to calculate mean importance of various factors across many individuals and for an individual level-intervention, it may be considered more important to know the reasons perceived by each individual as “top”. Further research to elucidate which approach to asking how food stores are selected is most informative for which populations and which purposes, and to consider whether there is something about incorporating both of the vantage points which can enrich understanding about the decision making processes surround food store selection.

It is important to note that, while proximity to home was an important reason for selecting one’s primary food store, there were several other reasons that were rated as important, if not more important, than proximity to home. Therefore, research that presumes that residents living near a food store will be likely shopping at this location [4-6] may be problematic. Future research may wish to directly assess the primary food store at which individuals shop and the amount of time that they have shopped at this store when examining the impact of primary food store on dietary intake and obesity, instead of assuming that the most proximal store is the one at which individuals shop.

This study had several strengths and limitations that should be considered when interpreting the results. First, the researchers were successful in recruiting a sample that was diverse in many ways, including race, age, educational background, and rurality. Although the majority of participants were female, it is likely that our sample is representative of primary household food shoppers; however, all of the participants belonged to households with at least two individuals, who did not follow a special diet requiring shopping at one store, and were available and interested in completing the questionnaire. Thus, the findings might not generalize to individuals who only shop for themselves, those with severe dietary restrictions, and those who did not have the time or interest in completing a questionnaire. In addition, despite achieving a sample diverse on several sociodemographic characteristics, all participants for the development and field testing of the questionnaire were from one relatively rural state; thus, the results are likely most generalizable to other rural states. However, the questionnaire was developed with the input of experts living in more urban areas and demonstrated some consistency with previous research in larger metropolitan areas [19,20] and could well be relevant for populations in more urban areas. Nonetheless, it will be important to examine the factors that are most important in food store choice in other communities (rural and urban). Furthermore, it is important to acknowledge the relatively small sample size in this study. In future research with a larger sample size, it will be interesting to examine whether reasons for choosing a store differ based on sociodemographic characteristics; with this information, it may be possible to tailor a food store intervention to reasons that are important for particular communities or sociodemographic groups. With a larger sample, it would also be possible examine the clustering of items among subgroups of individuals as well as the potential underlying domains of reasons for choosing a food store. Finally, it will be crucial to examine the reliability of the questionnaire in future research.

## Conclusions

Upon confirmation of these findings in other communities, knowing the primary reasons why individuals choose a food store may be beneficial in guiding policies and programs in making food environmental changes crafted to support dietary change and obesity prevention. In particular, based on the findings in the current study, future food store interventions may wish to focus on the availability, quality, variety, and prices of fruits, vegetable and meats, rather than a more global focus on more healthful food items (e.g., diet beverages, baked/low-fat chips) as has been done in many food store interventions [25-28]. Furthermore, our findings indicate that store characteristics including payment options, cleanliness, maintenance, safety, and opening hours of the store will be crucial factors in the potential success of such an intervention. Nonetheless, the sustainability of a healthy store environment will likely be greatly impacted by other factors including macro-level influences (e.g., food and agriculture policies) [29,30].

## Competing interests

The authors declare that they have no competing interests.

## Author contributions

RK conceived of the study and obtained funding, and participated in its design and coordination, drafted the manuscript, and performed the quantitative analyses. CS and MD participated in the study’s design and coordination. JM participated in the design of the study. DW conceived of the study and obtained funding, and participated in its design and coordination. All authors read and approved the final manuscript.

## Pre-publication history

The pre-publication history for this paper can be accessed here:

http://www.biomedcentral.com/1471-2458/13/586/prepub

## Supplementary Material

Additional file 1 Food Store Selection Questionnaire. Click here for file
