# Supplementary material for: There’s more to food store choice than proximity: a questionnaire development study
Source: BMC Public Health. 2013 Jun 17;13:586. doi: 10.1186/1471-2458-13-586 (PMC3686698; doi:10.1186/1471-2458-13-586)
Supplement: Additional file 1 — Food Store Selection Questionnaire. [file 1471-2458-13-586-S1.docx]

| **A. Below you will find reasons you might decide to go to a store to shop for food.**   - **To the right of each reason, please rate how important that reason is FOR YOU in choosing a store where you would want to shop for food.** - **Fill in the circle for the rating from "Not Important" to "Very Important" that best indicates how important that reason is to your choice of stores.**    - ***For example, if the freshness of the meat at the store is a very important reason for you to want to go to a store, you would fill in the circle for “Very Important.”*** - **There are no right or wrong answers. We are interested in your thoughts and opinions.** - **Please rate EACH reason.** | | | | | |
| --- | --- | --- | --- | --- | --- |
|  | **Not At All Important 1** | **Minimally Important 2** | **Somewhat Important 3** | **Moderately Important 4** | **Very Important 5** |
| **LOCATION** |  |  |  |  |  |
| 1. It's close to my work.  - Does not apply—I don’t work. | **○** | **○** | **○** | **○** | **○** |
| 1. It's close to my home. | **○** | **○** | **○** | **○** | **○** |
| 1. It's close to other stores where I shop. | **○** | **○** | **○** | **○** | **○** |
| 1. It’s close to public transportation such as a bus, train, or subway. | **○** | **○** | **○** | **○** | **○** |
| **PRODUCT AVAILABILITY AND QUALITY** |  |  |  |  |  |
| 1. I can buy organic/chemical-free foods. | **○** | **○** | **○** | **○** | **○** |
| 1. I can buy locally grown/raised foods. | **○** | **○** | **○** | **○** | **○** |
| 1. I think the fruits and vegetables are fresh and not bruised. | **○** | **○** | **○** | **○** | **○** |
| 1. I can choose from a wide variety of fruits and vegetables. | **○** | **○** | **○** | **○** | **○** |
| 1. I think the meat is fresh. | **○** | **○** | **○** | **○** | **○** |
| 1. I can buy the kinds of meat that I want. | **○** | **○** | **○** | **○** | **○** |
| 1. I can buy foods in bulk or large volumes. | **○** | **○** | **○** | **○** | **○** |
| 1. I can find the brands that I like. | **○** | **○** | **○** | **○** | **○** |
| 1. I can buy lower cost items like store brand items. | **○** | **○** | **○** | **○** | **○** |
| 1. The store has a good variety of healthy foods. | **○** | **○** | **○** | **○** | **○** |
| 1. I can buy the foods that my family needs for medical reasons (for example, low-salt or gluten-free foods). | **○** | **○** | **○** | **○** | **○** |
| 1. I can buy the foods that I eat for religious reasons (for example, kosher). | **○** | **○** | **○** | **○** | **○** |
| 1. I can buy non-food items I need (for example, clothing). | **○** | **○** | **○** | **○** | **○** |
| 1. The store has foods that are not going to quickly expire. | **○** | **○** | **○** | **○** | **○** |
| 1. The store has foods that I know how to prepare. | **○** | **○** | **○** | **○** | **○** |
|  | **Not At All Important 1** | **Minimally Important 2** | **Somewhat Important 3** | **Moderately Important 4** | **Very Important 5** |
| 1. The store has foods that I grew up eating. | **○** | **○** | **○** | **○** | **○** |
| **STORE CHARACTERISTICS** |  |  |  |  |  |
| 1. I like the size of the store. | **○** | **○** | **○** | **○** | **○** |
| 1. I like that the store has a butcher. | **○** | **○** | **○** | **○** | **○** |
| 1. I like that the store contains a pharmacy. | **○** | **○** | **○** | **○** | **○** |
| 1. I can complete my shopping quickly. | **○** | **○** | **○** | **○** | **○** |
| 1. I can easily find parking. | **○** | **○** | **○** | **○** | **○** |
| 1. I can always get a shopping cart/basket. | **○** | **○** | **○** | **○** | **○** |
| 1. I am familiar with the store and its layout. | **○** | **○** | **○** | **○** | **○** |
| 1. The aisles of the store are wide enough. | **○** | **○** | **○** | **○** | **○** |
| 1. I think the store is clean. | **○** | **○** | **○** | **○** | **○** |
| 1. I feel safe when I go there. | **○** | **○** | **○** | **○** | **○** |
| 1. The store is open when I like to go shopping. | **○** | **○** | **○** | **○** | **○** |
| 1. I am able to easily find the items on my shopping list. | **○** | **○** | **○** | **○** | **○** |
| 1. The store is well-maintained. | **○** | **○** | **○** | **○** | **○** |
| 1. The store is kept at a good temperature. | **○** | **○** | **○** | **○** | **○** |
| 1. The employees at the store quickly respond to my needs. | **○** | **○** | **○** | **○** | **○** |
| 1. The store delivers to my home. | **○** | **○** | **○** | **○** | **○** |
| 1. I am able to order my groceries from this store on the Internet. | **○** | **○** | **○** | **○** | **○** |
| 1. I like that the store has a bakery. | **○** | **○** | **○** | **○** | **○** |
| 1. The store is environmentally-conscious. | **○** | **○** | **○** | **○** | **○** |
| 1. The store treats their employees well (for example, pay and benefits). | **○** | **○** | **○** | **○** | **○** |
| 1. The store has disinfecting wipes for the grocery cart available. | **○** | **○** | **○** | **○** | **○** |
| 1. The store generally has enough cashiers open when I am shopping. | **○** | **○** | **○** | **○** | **○** |
| 1. The store generally has someone available to bag my groceries. | **○** | **○** | **○** | **○** | **○** |

|  | **Not At All Important 1** | **Minimally Important 2** | **Somewhat Important 3** | **Moderately Important 4** | **Very Important 5** |
| --- | --- | --- | --- | --- | --- |
| **COST AND PAYMENT** | **○** | **○** | **○** | **○** | **○** |
| 1. I think the store has low prices. | **○** | **○** | **○** | **○** | **○** |
| 1. The store accepts coupons. | **○** | **○** | **○** | **○** | **○** |
| 1. I think the store has good sales. | **○** | **○** | **○** | **○** | **○** |
| 1. I can get a discount by using the store’s shopper’s card. | **○** | **○** | **○** | **○** | **○** |
| 1. The store sends out a sale paper or advertisement that helps me plan what I will buy. | **○** | **○** | **○** | **○** | **○** |
| 1. The store has a payment option that works for me (that is, personal check, type of debit/credit card, purchase account). | **○** | **○** | **○** | **○** | **○** |

Which of the previous reasons are the two most important reasons for you in choosing in a food store (briefly indicate item and item number)?

1. _________________________________________________
2. _________________________________________________

Additional Comments Regarding Factors Important for Choosing Food Stores:

____________________________________________________________________________________________________________________________________________________________________________________________________________________________________________________________________________________________________________________________________________________________________________________________________________________________________________________________________________________________________________________________________________________________
